# Tert‐butyl hydroperoxide induces trabecular meshwork cells injury through ferroptotic cell death

**DOI:** 10.1002/ccs3.12050

**Published:** 2024-08-28

**Authors:** Xuejing Yan, Qian Liu, Shen Wu, Xiaowei Fan, Yufei Teng, Ningli Wang, Jingxue Zhang

**Affiliations:** ^1^ Beijing Institute of Ophthalmology Beijing Tongren Eye Center Beijing Tongren Hospital Capital Medical University Beijing Ophthalmology & Visual Sciences Key Laboratory Beijing China; ^2^ Beijing Institute of Brain Disorders Collaborative Innovation Center for Brain Disorders Capital Medical University Beijing China

**Keywords:** ferroptosis, glaucoma, oxidative stress, trabecular meshwork

## Abstract

Trabecular meshwork (TM) tissue has a crucial role in regulating aqueous humor circulation in the eye, thus maintaining normal intraocular pressure (IOP). TM dysfunction causes IOP elevation, which leads to glaucoma. To investigate biological changes in TM tissue in patients with glaucoma, we analyzed the mRNA expression microarray dataset, GSE27276. Gene ontology analysis indicated that redox microenvironment imbalance is among the main changes of TM tissue in patients with glaucoma. Subsequently, we induced oxidative stress in TM cells using the tert‐butyl hydroperoxide (tBHP) treatment, to generate in vivo and in vitro models, and conducted mRNA sequencing to identify genes with critical roles in maintaining the redox microenvironment balance. We found that the tBHP caused TM dysfunction in vivo, characterized by aqueous humor circulation resistance, IOP elevation, and TM cell death. Further, Kyoto Encyclopedia of Genes and Genomes pathway analysis showed that ferroptosis signaling was enriched in tBHP‐treated TM cells. Consistently, in vitro analyses showed that levels of reactive oxygen species, ferric ion, and malondialdehyde were increased after the tBHP treatment, indicating TM cell ferroptosis. Furthermore, inhibiting ferroptosis alleviated tBHP‐induced TM cell injury. This study provides new insights suggesting that inhibition of ferroptosis has potential as a treatment for glaucoma.

## Graphical abstract

Diagram illustrating tBHP‐induced ferroptosis in trabecular meshwork (TM) cells. Under oxidative stress (OS) conditions induced by tBHP, the intracellular Fe^2^⁺ concentration increases while GPX4 expression decreases, leading to ferroptosis in TM cells. Treatment with Deferiprone mitigates tBHP‐induced ferroptosis in TM cells.

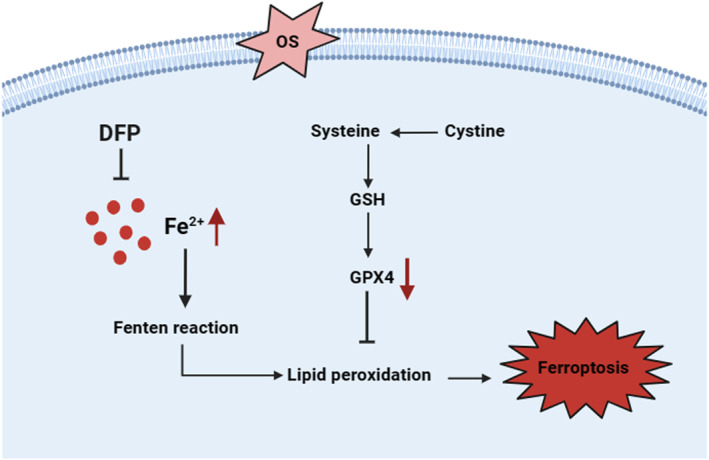

## 1 INTRODUCTION

Glaucoma is the most common irreversible eye disease leading to sight loss and is expected to affect 112 million people worldwide by 2040.^1^ Primary open angle glaucoma (POAG) is the most common form of glaucoma, and is characterized by progressive retinal ganglion cell (RGC) loss and visual impairment.^2^ Increased intraocular pressure (IOP) is a major risk factor for POAG, and reducing IOP is currently the only therapeutic strategy available for POAG treatment.^3^ The dynamic balance between aqueous humor production and outflow is a key factor in maintaining normal IOP, and trabecular meshwork (TM) tissue plays an important role in regulating aqueous humor circulation. Presently, surgery or drugs are the main methods used to promote aqueous humor efflux or reduce aqueous humor production; however, these treatments do not inhibit disease progression or prevent further visual impairment. Therefore, exploration of the mechanism underlying TM cell injury in glaucoma and identification of long‐term and lasting therapeutic strategies is of great significance.

Oxidative stress is a direct risk factor for increased IOP in patients with glaucoma, and antioxidants have great potential in the treatment of glaucoma.^4^, ^5^, ^6^ It is manifested as an imbalance of oxidation and antioxidant effects. Levels of antioxidant factors, such as malondialdehyde (MDA) and glutathione (GSH), are significantly reduced in the aqueous humor of patients with glaucoma, while oxidase activity is significantly increased,^7^, ^8^, ^9^ suggesting a decrease in overall antioxidant capacity in patients with glaucoma. TM is the tissue in the anterior chamber of the eye that is most sensitive to oxidative damage.^10^ Studies by our team and others have shown that OS is among the major changes in TM tissue in patients with glaucoma.^11^, ^12^ In addition, oxidation markers and lipid peroxidation products are significantly increased in TM cells from patients with glaucoma,^13^, ^14^, ^15^, ^16^ and the degree of visual field defect in patients with glaucoma is positively correlated with the degree of TM oxidative damage.^15^, ^17^ Together, these findings suggest that there is significant redox microenvironment imbalance in TM tissue of patients with glaucoma and that this imbalance is a key factor contributing to glaucoma development.

A balanced cellular redox microenvironment is a basic requirement for the normal development of various biological processes, while disruption of the intracellular redox equilibrium can lead to lipid peroxidation and eventually ferroptosis.^18^ Ferroptosis is a recently discovered form of iron‐dependent regulated cell death^18^ that functions in various physiological and pathological processes, including cancer,^19^ neurodegeneration,^20^ and ischemia‐reperfusion injury.^21^ Glutathione (GSH) is the major intracellular thiol antioxidant, and has key roles in cellular redox homeostasis by scavenging reactive oxygen species (ROS) and reactive nitrogen species,^22^ as well as being essential for ferroptosis. Other key molecules are associated with the regulation, synthesis, recycling, and utilization of GSH within cells, including SLC7A11, glutamate‐cysteine ligase (GCL), glutathione synthetase, and glutathione peroxidase 4 (GPX4).^23^

Iron has a dual function in ferroptosis, acting as both a catalytic cofactor for lipid peroxidation and a regulator of cellular antioxidant defense mechanisms.^24^ Various molecules that participate in iron metabolism have important functions in ferroptosis: transferrin receptor 1 (TFRC) is a membrane‐bound protein involved in iron uptake from transferrin (TF); and heme oxygenase 1 (HMOX1) catalyzes the degradation of heme into biliverdin, iron, and carbon monoxide. Further, excessive heme release and iron accumulation can promote OS and lipid peroxidation, leading to ferroptosis.

In recent years, the role of ferroptosis in the process of RGC cell injury in glaucoma has been gradually revealed. The glutamate receptor, N‐methyl‐D‐aspartate (NMDA) receptor, leads to RGC ferroptosis by regulating iron metabolism,^25^ and NMDA injection in the vitreous cavity can cause ferrous ion accumulation in the RGC and RGC cell loss 7 days after injection, while administration of an iron chelating agent can inhibit NMDA‐induced RGC damage and reduce ferrous ion accumulation and lipid peroxidation.^26^ Pathologically increased IOP can disrupt the iron metabolic balance of RGC and promote their ferroptosis.^27^ Hence, ferroptosis may contribute to RGC injury and lead to glaucoma development; however, no studies have investigated whether ferroptosis is involved in trabecular network injury in the context of glaucoma.

Here, we explored ferroptosis and changes in the transcriptome profile of tert‐butyl hydroperoxide (tBHP)‐treated human primary TM cells. Further, we investigated the main signaling pathways involved in response to OS. Our data indicate that inhibiting ferroptosis is a potential strategy for treatment of POAG.

## 2 MATERIALS AND METHODS

### 2.1 Data collection and analysis of differentially expressed genes (DEGs)

The GSE27276 dataset, which contained mRNA expression microarray data from 19 POAG and 17 normal control samples, was downloaded and analyzed using R packages. DEGs between POAG and control samples were visualized using heatmaps and volcano plots and gene ontology (GO) analysis conducted in R packages.

### 2.2 Animals

This study was approved and monitored by the Institutional Animal Care and Use Committee of the Capital Medical University of Beijing (IACUC; AEEI‐2018‐198), and conformed to the National Institute of Health Guide for the Care and Use of Laboratory Animals, as well as the Association for Research in Vision and Ophthalmology Statement for the Use of Animals in Ophthalmic and Vision Research.

C57BL/6J mice (8 weeks old) were purchased from Zhejiang Vital River Experimental Animal Technology Co. Ltd (Charles River Lab) for use in this study, and housed at humidity 40%–60% with 12:12 h light‐dark cycle.

### 2.3 Topical administration of tBHP in the eye

C57BL/6J mouse were administered 0.1% tBHP (Sigma‐Aldrich, Inc.) by eye drop twice daily (9:00–10:00 a.m. and 5:00–6:00 p.m.) diluted in sterile PBS, as described in invention patent CN 115336553; the same volume of sterile PBS served as a control. Drug treatment was continued for 16 days.

### 2.4 Evaluation of aqueous humor dynamics

Gadolinium magnetic resonance imaging was applied to evaluate aqueous humor dynamics as previously described.^28^, ^29^ Mice were anesthetized with 2% isoflurane (Shandong ante animal husbandry technology) and respiration rate monitored using a small pneumatic pillow. Baseline measurements were initially obtained, then mice were intraperitoneally injected with 0.3 mmol/kg Gd‐diethylenetriamine pentametric acid. Images were acquired every 7 min for 42 min. Signal intensity in areas of interest was analyzed using the ImageJ software; all measurements were normalized to the baseline image.

### 2.5 Intraocular pressure measurement

Animals were anesthetized with 5% isoflurane for 3 min, and we slightly press the measurement button to make sure the tip of the probe contact the central cornea vertically using TonoLab rebound tonometry (Icare). The probe is 1–4 mm from corneal surface. For each IOP measurement, the probe strikes the cornea five times consecutively and the tonometer displayed the average of 5 measurements as the resulting value. We repeated the measurements three times and then averaged the three resulting values. IOP was measured between 3:00 and 5:00 p.m.

### 2.6 Slit lamp examination

Slit‐lamp (BX‐900, HAAG‐STREIT AG) examination was used to evaluate the anterior segment, including iris, lens and cornea. Photographs were captured using a digital camera using consistent parameters throughout. Briefly, mice was anesthetized with 5% isoflurane for 3 min and gently open the mouse's eyelids. Position the slit lamp microscope in front of the mouse's eye, and adjust the beam width and angle to illuminate the anterior chamber angle. Finally, images were taken from the optimum perspectives.

### 2.7 Immunostaining

Mice were euthanized and enucleated eyes immersed in 4% paraformaldehyde (Beyotime) overnight, after which the enucleated eyes were dehydrated with gradient ethanol and embedded in paraffin. Tissues were sliced into 5 μm thick sections, which were deparaffinized using xylene and gradient ethanol, then immersed in citric buffer, microwaved for antigen retrieval, and cooled down to room temperature. Subsequently, sections were blocked with 5% BSA (Solarbio) for 30 min at room temperature, incubated with primary antibody (anti‐α‐SMA) overnight. After washing with PBS (Servicebio) three times, sections were incubated with Alexa Fluor™ 546‐conjugated goat anti‐rabbit IgG (Thermo Fisher) for 2 h. Finally, nuclei were stained with DAPI (Sigma‐Aldrich, Inc.). Images were acquired using a confocal microscope (Leica).

### 2.8 TUNEL assay

TUNEL assays were performed using a TUNEL Apoptosis Detection Kit (Vazyme). Paraffin sections were deparaffinized with xylene and gradient ethanol. Proteinase K was used for tissue digestion, then 100 μL 1 × Equilibration Buffer added to fully cover the sample area and allowed to equilibrate at room temperature for 30 min. Subsequently, samples were incubated with 50 μL TdT Buffer for 1 h at 37°C, washed with PBS, then stained with DAPI (Sigma‐Aldrich, Inc.). Photographs were taken using a confocal microscope (Leica).

### 2.9 Cell culture

Human primary TM cells were obtained from ScienCell (Carlsbad, CA, United States) and grown in TMCM medium (ScienCell), supplemented with 2% fetal bovine serum (ScienCell), 1% penicillin/streptomycin solution (ScienCell), and 1% TM cell growth supplement (ScienCell). All cells used for experiments were cultured for <6 passages.

### 2.10 Drug treatment

tBHP was diluted in culture medium at various concentrations for treatment of human TM cells for 24 h before subsequent analyses.

### 2.11 Measurement of intracellular ferric ion, MDA, and ROS levels

Intracellular iron and MDA levels were measured using iron and MDA assay kits (Nanjing Jiancheng Bioengineering Institute), according to the manufacturer's instructions. Intracellular ROS levels were measured using a DCFA‐DA detection kit (Beyotime).

### 2.12 Cell viability detection

TM cells with or without tBHP treatment were seeded in triplicate into 96‐well plates at 1000 cells per well for 24 h. 10 μL of CCK‐8 solution (DOJINDO) was added to each well and then samples incubated for 2 h, followed by cell viability detection by measuring absorbance at 450 nm using a microplate reader.

### 2.13 Apoptosis detection

TM cells with or without tBHP treatment were digested using 0.25% trypsin (Gibco) for 1 min, then centrifuged for 5 min at 300 g, and washed twice with pre‐cooled PBS. Cells were resuspended in 1 × binding buffer at a final concentration of 2 × 10^6^/mL. Cells were then immediately stained with PI/Annexin V‐FITC (Yeasen) and subjected to apoptosis detection by flow cytometry.

### 2.14 mRNA sequencing (RNA‐seq)

After tBHP treatment for 24 h, cells were washed with 1 × PBS and TRIzol reagent (Invitrogen) added for total RNA extraction and purification, according to the instructions. RNA samples (2 μg) were used as input material for preparation of sequencing libraries using a NEBNext Ultra^TM^ RNA Library Prep Kit for Illumina (NEB), according to the manufacturer's instructions. After cluster generation, prepared libraries were sequenced on a Illumina Hiseq 4000 platform, to generate paired‐end 150‐bp reads. After quality control, high quality reads were mapped to the reference genome sequence. Only reads with a perfect match or a single base pair mismatch to the reference genome were further analyzed and annotated.

Differential expression analysis was performed using the DESeq package in R (1.10.1). Resulting *p* values were adjusted using the Benjamini–Hochberg approach, to control the false discovery rate. Genes with adjusted *p* < 0.05 by DESeq were assigned as differentially expressed.

Functional analysis and Kyoto Encyclopedia of Genes and Genomes (KEGG) pathway analysis of the annotated list of differentially expressed genes (DEGs) were conducted using the GOseq R package and KOBAS software.

### 2.15 Quantitative real‐time polymerase chain reaction analysis

Cells were harvested using TRIzol reagent (Invitrogen), and RNA extracted according to the manufacturer's instructions. Total RNA (500 ng) was reverse transcribed using HiScriptII Q RT SuperMix for qPCR (Vazyme). cDNA was quantified using the ChamQ^TM^ Universal SYBR qPCR Master Mix (Vazyme) on a Bio‐Rad machine. Primer sequences were list in Table S1. *β*‐actin served as the internal control.

### 2.16 Western blot analysis

TM cells were lysed in RIPA buffer (Beyotime) for 30 min on ice. Protein concentrations were quantified using a BCA kit (Thermo Fisher). Total protein aliquots (40 μg) were subjected to 10% sodium dodecyl sulfate‐polyacrylamide gel electrophoresis and transferred to PVDF membrane (Millipore). Membranes were blocked in 5% non‐fat milk (Yeasen) for 2 h, then incubated with primary antibodies at 4°C overnight. After washing three times with 0.1% TBST, membranes were incubated with appropriate secondary antibodies. Signals were detected using a Bio‐RAD imaging system. The detailed information of primary antibodies was listed in Table S2. The secondary antibodies were as follows: HRP‐linked anti‐mouse IgG (Cell Signaling) and HRP‐linked anti‐rabbit IgG (Cell Signaling).

### 2.17 Statistical analysis

Statistical data are reported as mean ± standard error of the mean of at least three independent biological repeats. Statistical analyses were performed using the GraphPad Prism software. Student's *t*‐test was used to compare two groups and one‐way ANOVA analysis of variance applied for comparisons of more than two groups. *p* < 0.05 was considered significant.

## 3 RESULTS

### 3.1 tBHP induced TM dysfunction and elevated IOP

**FIGURE 1:**
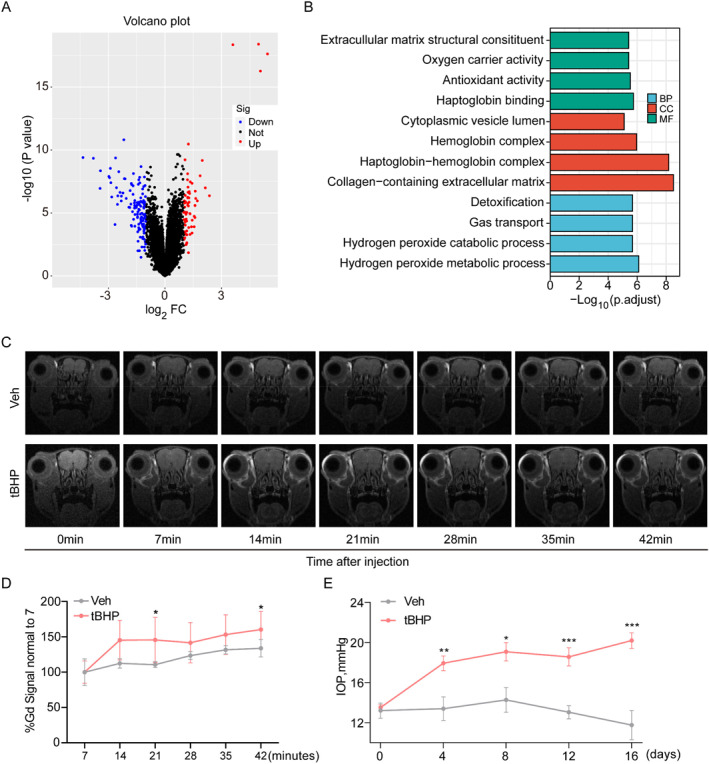
Oxidative stress correlates with trabecular meshwork injury. (A). Volcano plot of differentially expressed genes (DEGs) between POAG and control samples in the GSE27276 dataset. (B). Gene ontology analysis of DEGs from GSE27276. (C). Gadolinium‐enhanced magnetic resonance images from vehicle (*n* = 4) and tBHP‐treated mice (*n* = 6) showing resistance of aqueous humor circulation. (D). Gd signal was significantly increased in tBHP‐treated mice by pixel intensity evaluation in the anterior chamber angle of (C) using Image J software. E. Intraocular pressure measurement in vehicle (*n* = 8) and tBHP‐treated mice (*n* = 8). Statistical analyses were determined using Student's *t*‐test. *p < 0.05; **p < 0.01; ***p < 0.001.

We analyzed the GSE27276 dataset and identified 97 DEGs between POAG and control samples, among which 32 and 118 transcripts were up‐ and down‐regulated in POAG samples, respectively (Figure 1A). GO analysis showed that DEGs were significantly enriched for redox related genes (Figure 1B), including those encoding proteins with oxygen carrier and antioxidant activities, and involved in hydrogen peroxide catabolic processes, suggesting that there may be redox microenvironment imbalance in the POAG TM tissue.

**FIGURE 2:**
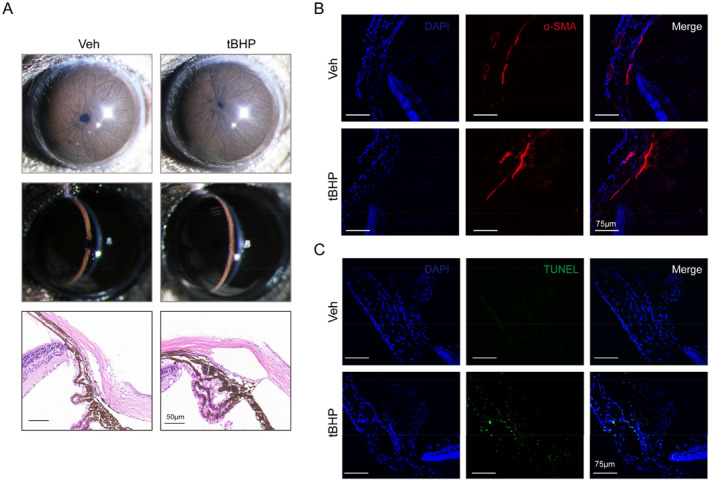
Tert‐butyl hydroperoxide (tBHP) has no effect on anterior chamber angle structure but induces trabecular meshwork (TM) stiffness, fibrosis and apoptosis. (A). Slit lamp examination (up) and Hematoxylin and Eosin (HE) staining (down) of vehicle (*n* = 3) and tBHP‐treated mice (*n* = 3), showing normal anterior chamber structure but TM stiffness. (B and C), Representative images of α‐SMA (B) and TUNEL (C) staining in TM sections from vehicle (*n* = 3) and tBHP‐treated mice (*n* = 3), indicating TM fibrosis and apoptosis.

To confirm whether OS in TM tissues causes TM dysfunction and IOP elevation, we administered tBHP to the eyes of mice, and found that aqueous humor circulation was inhibited (Figure 1C,D) and IOP elevated (Figure 1E) after tBHP treatment. Moreover, tBHP had no effect on anterior angle structure (Figure 2A), but promoted TM stiffness (Figure 2A), fibrosis (Figure 2B) and apoptosis (Figure 2C) in TM tissue. These results indicate that tBHP treatment can induce TM tissue injury.

### 3.2 tBHP treatment induced TM cell death

**FIGURE 3:**
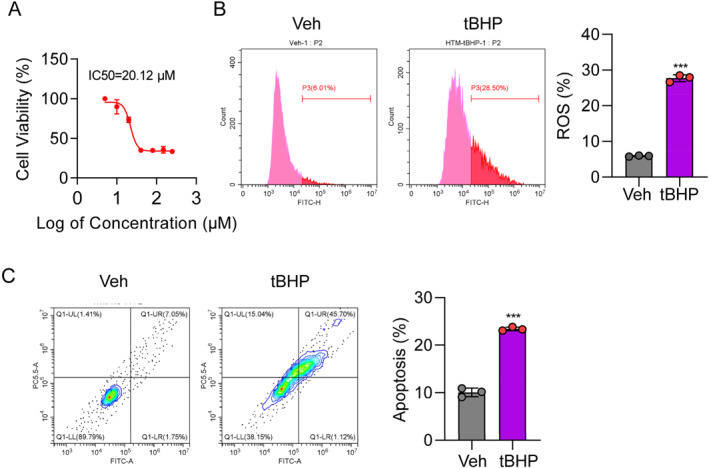
Tert‐butyl hydroperoxide (tBHP) induces trabecular meshwork (TM) cell injury. (A). Cell viability analysis of TM cells with (*n* = 5) or without tBHP treatment (*n* = 5) by CCK8 assay. (B). Reactive oxygen species detection in TM cells with (*n* = 3) or without tBHP treatment (*n* = 3) by flow cytometry. (C). Detection of TM cell apoptosis with (*n* = 3) or without tBHP treatment (*n* = 3) by flow cytometry. Statistical analyses was performed using Student's *t*‐test. ***p < 0.001.

To investigate the mechanism involved in TM cell OS, we treated TM cells with tBHP at various concentrations to induce injury and then assessed cell viability. TM cell viability was significantly decreased to approximately 50% after treatment with 30 μM tBHP (Figure 3A); therefore, this concentration was subsequently used to treat TM cells and generate an OS model. ROS level (Figure 3B) and cell death rate (Figure 3C) were both markedly increased in response to tBHP administration. These results indicate that tBHP can induce TM cell death.

### 3.3 GO and KEGG analysis of DEGs in tBHP‐treated TM cells

**FIGURE 4:**
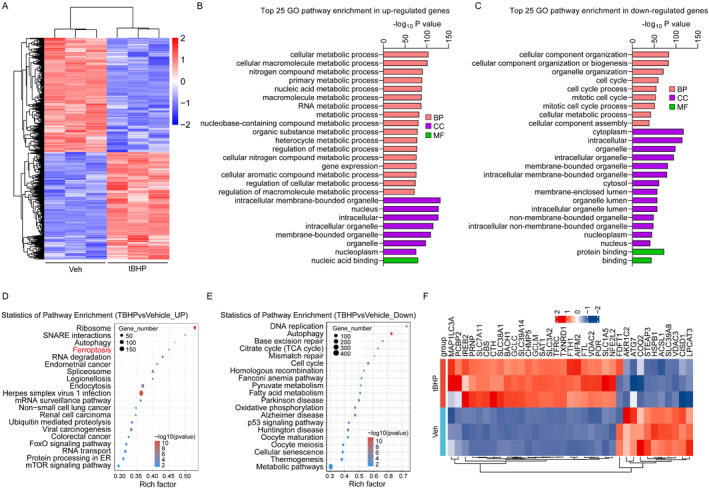
Gene ontology (GO) and Kyoto Encyclopedia of Genes and Genomes (KEGG) analysis of differentially expressed genes (DEGs) in trabecular meshwork (TM) cells after tert‐butyl hydroperoxide (tBHP) treatment. (A). Heatmap of DEGs in TM cells with or without tBHP treatment. B–C, GO analysis showing the top 25 pathways enriched for genes up‐regulated (B) and down‐regulated (C) in tBHP‐treated TM cells. D–E, KEGG pathway enrichment analysis of genes up‐regulated (D) and down‐regulated (E) in tBHP‐treated TM cells. F. Heatmap of ferroptosis‐related genes in TM cells with or without tBHP treatment.

To further investigate genes important in protecting TM cells from OS, we next performed mRNA sequencing of tBHP‐treated TM cells. After mRNA sequencing, we screened DEGs using the following criteria: fold‐change ≥1.5 or ≤0.66 and *p* < 0.05. Figure 4A shows a heatmap of DEGs in TM cells without or with tBHP treatment, demonstrating that the mRNA expression profile of TM cells was markedly changed in response to tBHP. GO analysis of DEGs indicated that tBHP induced clear upregulation of genes correlated with cellular metabolic processes (Figure 4B) and a significant downregulation of genes involved in cellular component organization and the cell cycle (Figure 4C). Further, KEGG pathway enrichment analysis to investigate how TM cells responded to OS indicated that up‐regulated genes altered on tBHP treatment were enriched for pathways including (but not limited to) autophagy, ferroptosis, and the mTOR signaling pathway (Figure 4D), whereas pathways enriched for down‐regulated genes included (but were not limited to) autophagy, fatty acid metabolism, oxidative phosphorylation, and cellular senescence (Figure 4E). Further, gene set enrichment analysis showed that ferroptosis was associated with tBHP‐induced TM cell death (Figure 4F). As shown in the heatmap in Figure 4F, the transcription profile of ferroptosis‐related genes was markedly altered in the tBHP treatment group relative to that in the vehicle control group. These results indicate that tBHP can induce ferroptosis of TM cells.

### 3.4 Inhibiting ferroptosis attenuates tBHP‐induced TM cell injury

**FIGURE 5:**
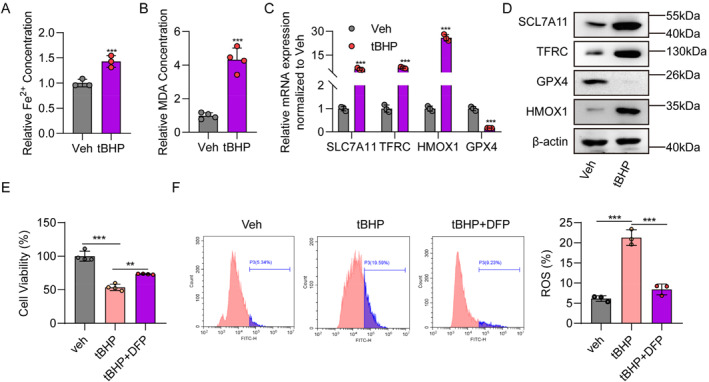
Deferiprone (DFP) effectively inhibits trabecular meshwork (TM) cell ferroptosis induced by tert‐butyl hydroperoxide (tBHP). (A). Measurement of ferric ion concentrations in TM cells with (*n* = 3) or without tBHP (*n* = 3) treatment. (B). Measurement of malondialdehyde concentrations in TM cells with (*n* = 3) or without tBHP (*n* = 3) treatment. (C). mRNA levels of *SLC7A11*, *TFRC*, *HMOX1*, and *GPX4* analyzed by quantitative real‐time polymerase chain reaction in TM cells with (*n* = 3) or without tBHP (*n* = 3) treatment. (D). Protein levels of SLC7A11, TFRC, HMOX1, and GPX4 analyzed by western blot in TM cells with (*n* = 3) or without tBHP (*n* = 3) treatment. (E). Cell viability analysis of vehicle (*n* = 4), tBHP (*n* = 4) and tBHP + DFP (*n* = 4) group determined by CCK8 assay. (F). ROS levels of vehicle (*n* = 3), tBHP (*n* = 3) and tBHP + DFP (*n* = 3) group determined by flow cytometry. The Student's *t*‐test was used to compare two groups and one‐way ANOVA analysis was applied for comparisons of more than two groups. *p* < 0.05 was considered significant. **p < 0.01; ***p < 0.001.

To further confirm that ferroptosis is involved in tBHP‐induced TM cell impairment, we next detected ferric ion and MDA concentrations. Cellular ferric ion and MDA levels were both significantly increased in TM cells after tBHP treatment (Figure 5A,B). Moreover, the mRNA and protein levels of ferroptosis driver molecules, including TFRC, and HMOX1, were all up‐regulated in the tBHP treatment group (Figure 5C,D), whereas the mRNA and protein levels of the ferroptosis suppressor, GPX4, were down‐regulated (Figure 5C,D). Surprisingly, both the mRNA and protein levels of SLC7A11, the ferroptosis suppressor, were markedly increased in the tBHP treatment group (Figure 5C,D). Further, we used deferiprone (DFP) to chelate free iron and found that it attenuated the tBHP‐induced inhibition of cell viability (Figure 5E) and ROS levels (Figure 5F). The results demonstrate that ferroptosis is correlated with tBHP‐induced TM cell damage.

## 4 DISCUSSION

The route via TM accounts for 85% of aqueous humor outflow, demonstrating the essential role of this tissue in regulating aqueous humor circulation.^30^ TM cell numbers are reported to be decreased in POAG^31^; however, the pathological role of TM tissue and the mechanism underlying TM impairment in POAG is largely unknown. In this study, we analyzed mRNA sequencing data from the GSE27276 dataset and found that antioxidant capacity was reduced in patients with POAG. Next, we applied tBHP to induce an in vivo OS model, and found that fibrosis‐related protein expression and cell death were increased in TM tissue after tBHP treatment. Moreover, tBHP promoted TM cell ferroptosis. Further, genome‐wide transcriptome analysis of TM cells after tBHP induction revealed that TM cell impairment may be attributable to abnormal GSH and iron metabolism. Finally, inhibiting ferroptosis alleviated the TM cell damage induced by tBHP.

A previous study by our team showed that OS is among the main biological changes in POAG TM tissue.^12^ OS represents an imbalance between ROS production and antioxidant defense mechanism and can cause extracellular matrix accumulation, mitochondrial damage, and chronic inflammatory responses in TM cells.^32^ Such TM dysfunction can then lead to disturbance of aqueous humor circulation, increased IOP, and glaucoma. Consistently, the findings of the present study indicate that OS damage to TM cells in vivo is characterized by the presence of increased apoptotic cells and fibrosis.

Chronic OS is a main driver of lipid peroxidation, accumulation of which is a factor that initiates the ferroptosis cascade. Iron is a key mediator of ferroptosis, as it promotes lipid peroxidation through the Fenton reaction, leading to cell membrane damage. TFRC is necessary for cellular iron uptake via receptor‐mediated endocytosis and TFRC upregulation promotes ferroptosis,^33^ while SLC40A1 participates in cellular iron efflux. HMOX1 also plays a crucial role in iron metabolism by catalyzing the breakdown of heme into biliverdin, carbon monoxide (CO), and iron, thereby contributing to cellular iron homeostasis, and can promote ferroptosis by inducing iron overload.^34^ In our study, we detected increased TFRC and HMOX1 expression and increased intracellular iron levels in TM cells after the tBHP treatment; therefore, we conclude that tBHP can promote TM cell ferroptosis by inducing abnormal iron metabolism.

GSH plays a crucial role in scavenging ROS and lipid peroxides, thereby protecting cells from oxidative damage that can trigger ferroptosis. SLC7A11, the main component of the cystine/glutamate antiporter system, provides L‐cystine for maintenance of redox balance by preserving intracellular glutathione levels, which is essential for protection of cells from OS. NRF2 was reported to inhibit ferroptosis by regulation of SLC7A11 in an acute lung injury model^35^; however, in our study, SLC7A11 expression levels were significantly up‐regulated following tBHP treatment. We hypothesize that the oxidized environment triggered by tBHP administration induced SLC7A11 to import GSH to counteract further damage.

Moreover, GPX4 activity helps preserve intracellular GSH levels by reducing lipid peroxides and preventing OS, while inhibition or depletion of GPX4 can lead to decreased GSH levels, compromising cellular antioxidant defenses, and thus inducing ferroptosis.^36^ In our study, GPX4 expression levels were significantly down‐regulated in tBHP‐induced TM cells. We conclude that ferroptosis may be a consequence of decreased antioxidant capacity.

Targeting ferroptosis has potential for application in research into therapeutic targets in disease.^18^ Inhibiting ferroptosis improves dyskinesia in mouse models of Parkinson's disease. Two iron chelators, deferoxamine and DFP, have been included in Phase II clinical trials for the treatment of neuronal damage caused by Parkinson's disease, dementia, intracranial hemorrhage, and ischemic stroke.^37^ Hence, targeting ferroptosis may also be a promising strategy for treatment of glaucoma.

In summary, we established both in vivo and in vitro models of TM OS to facilitate investigation of the mechanism underlying TM impairment in POAG. Furthermore, we found that tBHP can induce TM cell ferroptosis by influencing both iron and GSH metabolism. This is the first study connecting ferroptosis with TM cell injury and our data indicate that targeting ferroptosis has potential as a strategy to treat glaucoma.

While this study provides valuable insights into mechanism of TM injury, there are several limitations that should be acknowledged. Firstly, due to the lack of clinical samples, this study was unable to validate the findings in glaucoma patients. Secondly, in future studies, we should confirm the results in more strains of human primary TM cells. Finally, the detailed mechanism of TM cells ferroptosis should be investigated. Despite these limitations, the study provides important contributions to the understanding of TM dysfunction and highlights areas for future research.

## AUTHOR CONTRIBUTIONS

Jingxue Zhang conceived and designed the study. Xuejing Yan conducted cell and animal experiments, performed bioinformatics analysis, and draft the manuscript. Qian Liu, Shen Wu, and Xiaowei Fan assisted the experiments. Yufei Teng and Ningli Wang assisted bioinformatics analysis. All authors read and critically revised the manuscript for intellectual content and approved the final manuscript.

## CONFLICT OF INTEREST STATEMENT

The authors declare that they have no competing interests.

## ETHICS STATEMENT

This study was approved and monitored by the Institutional Animal Care and Use Committee of the Capital Medical University of Beijing (IACUC; AEEI‐2018‐198).

## Supporting information

Table S1

Table S2

## Data Availability

All the data and materials supporting the conclusion of this study have been included within the article.

## References

[1] Tham, Y.‐C. , X. Li , T. Y. Wong , H. A. Quigley , T. Aung , and C.‐Y. Cheng . 2014. “Global Prevalence of Glaucoma and Projections of Glaucoma Burden through 2040: A Systematic Review and Meta‐Analysis.” Ophthalmology 121(11): 2081–2090. 10.1016/j.ophtha.2014.05.013. 24974815

[2] Stein, J. D. , A. P. Khawaja , and J. S. Weizer . 2021. “Glaucoma in Adults‐Screening, Diagnosis, and Management: A Review.” JAMA 325(2): 164–174. 10.1001/jama.2020.21899. 33433580

[3] Weinreb, R. N. , T. Aung , and F. A. Medeiros . 2014. “The Pathophysiology and Treatment of Glaucoma: A Review.” JAMA 311(18): 1901–1911. 10.1001/jama.2014.3192. 24825645 PMC4523637

[4] Amankwa, C. E. , O. Young , B. DebNath , S. R. Gondi , R. Rangan , D. Z. Ellis , G. Zode , D. L. Stankowska , and S. Acharya . 2023. “Modulation of Mitochondrial Metabolic Parameters and Antioxidant Enzymes in Healthy and Glaucomatous Trabecular Meshwork Cells with Hybrid Small Molecule SA‐2.” International Journal of Molecular Sciences 24(14): 11557. 10.3390/ijms241411557. 37511316 PMC10380487

[5] Amankwa, C. E. , B. Kodati , N. Donkor , and S. Acharya . 2023. “Therapeutic Potential of Antioxidants and Hybrid TEMPOL Derivatives in Ocular Neurodegenerative Diseases: A Glimpse into the Future.” Biomedicines 11(11): 2959. 10.3390/biomedicines11112959. 38001960 PMC10669210

[6] Amankwa, C. E. , S. R. Gondi , A. Dibas , C. Weston , A. Funk , T. Nguyen , K. T. Nguyen , D. Z. Ellis , and S. Acharya . 2021. “Novel Thiol Containing Hybrid Antioxidant‐Nitric Oxide Donor Small Molecules for Treatment of Glaucoma.” Antioxidants 10(4): 575. 10.3390/antiox10040575. 33917924 PMC8068288

[7] Fahmy, H. M. , E. Saad , N. M. Sabra , A. A. El‐Gohary , F. F. Mohamed , and M. H. Gaber . 2018. “Treatment Merits of Latanoprost/Thymoquinone ‐ Encapsulated Liposome for Glaucomatus Rabbits.” International Journal of Pharmacy 548(1): 597–608. 10.1016/j.ijpharm.2018.07.012. 29997042

[8] Carrim, N. , T. G. Walsh , A. Consonni , M. Torti , M. C. Berndt , and P. Metharom . 2014. “Role of Focal Adhesion Tyrosine Kinases in GPVI‐dependent Platelet Activation and Reactive Oxygen Species Formation.” PLoS One 9(11): e113679. 10.1371/journal.pone.0113679. 25415317 PMC4240642

[9] Su, J. , and M. Huang . 2018. “Etidronate Protects Chronic Ocular Hypertension Induced Retinal Oxidative Stress and Promotes Retinal Ganglion Cells Growth through IGF‐1 Signaling Pathway.” European Journal of Pharmacology 841: 75–81. 10.1016/j.ejphar.2018.10.002. 30326214

[10] Izzotti, A. , S. C. Saccà , M. Longobardi , and C. Cartiglia . 2009. “Sensitivity of Ocular Anterior Chamber Tissues to Oxidative Damage and its Relevance to the Pathogenesis of Glaucoma.” Investigative Ophthalmology & Visual Science 50(11): 5251–5258. 10.1167/iovs.09-3871. 19516005

[11] Zhao, J. , S. Wang , W. Zhong , B. Yang , L. Sun , and Y. Zheng . 2016. “Oxidative Stress in the Trabecular Meshwork (Review).” International Journal of Molecular Medicine 38(4): 995–1002. 10.3892/ijmm.2016.2714. 27572245

[12] Wu, J. , C. Lin , C. Yang , L. Pan , H. Liu , S. Zhu , S. Wei , et al. 2023. “Identification and Validation of Key Biomarkers and Potential Therapeutic Targets for Primary Open‐Angle Glaucoma.” Science China Life Sciences 66(12): 2837–2850. 10.1007/s11427-022-2344-5. 37610681

[13] Hondur, G. , E. Göktas , X. Yang , L. Al‐Aswad , J. D. Auran , D. M. Blumberg , G. A. Cioffi , et al. 2017. “Oxidative Stress‐Related Molecular Biomarker Candidates for Glaucoma.” Investigative Ophthalmology & Visual Science 58(10): 4078–4088. 10.1167/iovs.17-22242. 28820925 PMC5685420

[14] He, Y. , K. W. Leung , Y.‐H. Zhang , S. Duan , X.‐F. Zhong , R.‐Z. Jiang , Z. Peng , J. Tombran‐Tink , and J. Ge . 2008. “Mitochondrial Complex I Defect Induces ROS Release and Degeneration in Trabecular Meshwork Cells of POAG Patients: Protection by Antioxidants.” Investigative Ophthalmology & Visual Science 49(4): 1447–1458. 10.1167/iovs.07-1361. 18385062

[15] Izzotti, A. , S. C. Saccà , C. Cartiglia , and S. De Flora . 2003. “Oxidative Deoxyribonucleic Acid Damage in the Eyes of Glaucoma Patients.” Americas Journal of Medicine 114(8): 638–646. 10.1016/s0002-9343(03)00114-1. 12798451

[16] Tabak, S. , S. Schreiber‐Avissar , and E. Beit‐Yannai . 2021. “Crosstalk between MicroRNA and Oxidative Stress in Primary Open‐Angle Glaucoma.” International Journal of Molecular Sciences 22(5): 2421. 10.3390/ijms22052421. 33670885 PMC7957693

[17] Saccà, S. C. , A. Pascotto , P. Camicione , P. Capris , and A. Izzotti . 2005. “Oxidative DNA Damage in the Human Trabecular Meshwork: Clinical Correlation in Patients with Primary Open‐Angle Glaucoma.” Archives of Ophthalmology 123(4): 458–463. 10.1001/archopht.123.4.458. 15824217

[18] Stockwell, B. R. 2022. “Ferroptosis Turns 10: Emerging Mechanisms, Physiological Functions, and Therapeutic Applications.” Cell. 185(14): 2401–2421. 10.1016/j.cell.2022.06.003. 35803244 PMC9273022

[19] Zhao, L. , X. Zhou , F. Xie , L. Zhang , H. Yan , J. Huang , C. Zhang , F. Zhou , J. Chen , and L. Zhang . 2022. “Ferroptosis in Cancer and Cancer Immunotherapy.” Cancer Communications 42(2): 88–116. 10.1002/cac2.12250. 35133083 PMC8822596

[20] Lane, D. J. R. , B. Metselaar , M. Greenough , A. I. Bush , and S. J. Ayton . 2021. “Ferroptosis and NRF2: An Emerging Battlefield in the Neurodegeneration of Alzheimer's Disease.” Essays in Biochemistry 65(7): 925–940. 10.1042/ebc20210017. 34623415

[21] Pan, Y. , X. Wang , X. Liu , L. Shen , Q. Chen , and Q. Shu . 2022. “Targeting Ferroptosis as a Promising Therapeutic Strategy for Ischemia‐Reperfusion Injury.” Antioxidants 11(11): 2196. 10.3390/antiox11112196. 36358568 PMC9686892

[22] Ursini, F. , and M. Maiorino . 2020. “Lipid Peroxidation and Ferroptosis: The Role of GSH and GPx4.” Free Radical Biology and Medicine 152: 175–185. 10.1016/j.freeradbiomed.2020.02.027. 32165281

[23] Bjørklund, G. , M. Peana , M. Maes , M. Dadar , and B. Severin . 2021. “The Glutathione System in Parkinson's Disease and its Progression.” Neuroscience & Biobehavioral Reviews 120: 470–478. 10.1016/j.neubiorev.2020.10.004. 33068556

[24] Fang, X. , H. Ardehali , J. Min , and F. Wang . 2023. “The Molecular and Metabolic Landscape of Iron and Ferroptosis in Cardiovascular Disease.” Nature Reviews Cardiology 20(1): 7–23. 10.1038/s41569-022-00735-4. 35788564 PMC9252571

[25] Chen, Y. , R. S. Khan , A. Cwanger , Y. Song , C. Steenstra , S. Bang , J. H. Cheah , et al. 2013. “Dexras1, a Small GTPase, Is Required for Glutamate‐NMDA Neurotoxicity.” Journal of Neuroscience 33(8): 3582–3587. 10.1523/jneurosci.1497-12.2013. 23426685 PMC3711661

[26] Sakamoto, K. , T. Suzuki , K. Takahashi , T. Koguchi , T. Hirayama , A. Mori , T. Nakahara , H. Nagasawa , and K. Ishii . 2018. “Iron‐chelating Agents Attenuate NMDA‐Induced Neuronal Injury via Reduction of Oxidative Stress in the Rat Retina.” Experimental Eye Research 171: 30–36. 10.1016/j.exer.2018.03.008. 29530811

[27] Yao, F. , J. Peng , E. Zhang , D. Ji , Z. Gao , Y. Tang , X. Yao , and X. Xia . 2023. “Pathologically High Intraocular Pressure Disturbs Normal Iron Homeostasis and Leads to Retinal Ganglion Cell Ferroptosis in Glaucoma.” Cell Death & Differentiation 30(1): 69–81. 10.1038/s41418-022-01046-4. 35933500 PMC9883496

[28] Ho, L. C. , I. P. Conner , C.‐W. Do , S.‐G. Kim , E. X. Wu , G. Wollstein , J. S. Schuman , and K. C. Chan . 2014. “In Vivo Assessment of Aqueous Humor Dynamics upon Chronic Ocular Hypertension and Hypotensive Drug Treatment Using Gadolinium‐Enhanced MRI.” Investigative Ophthalmology & Visual Science 55(6): 3747–3757. 10.1167/iovs.14-14263. 24764067 PMC4062398

[29] Nair, K. S. , C. Srivastava , R. V. Brown , S. Koli , H. Choquet , H. S. Kang , Y.‐M. Kuo , et al. 2021. “GLIS1 Regulates Trabecular Meshwork Function and Intraocular Pressure and Is Associated with Glaucoma in Humans.” Nature Communications 12(1): 4877. 10.1038/s41467-021-25181-7. PMC8361148 34385434

[30] Jonas, J. B. , T. Aung , R. R. Bourne , A. M. Bron , R. Ritch , and S. Panda‐Jonas . 2017. “Glaucoma.” Lancet 390(10108): 2183–2193. 10.1016/s0140-6736(17)31469-1. 28577860

[31] Carreon, T. A. , G. Edwards , H. Wang , and S. K. Bhattacharya . 2017. “Segmental Outflow of Aqueous Humor in Mouse and Human.” Experimental Eye Research 158: 59–66. 10.1016/j.exer.2016.08.001. 27498226 PMC5290258

[32] Baudouin, C. , M. Kolko , S. Melik‐Parsadaniantz , and E. M. Messmer . 2021. “Inflammation in Glaucoma: From the Back to the Front of the Eye, and beyond.” Progress in Retinal and Eye Research 83: 100916. 10.1016/j.preteyeres.2020.100916. 33075485

[33] Yi, L. , Y. Hu , Z. Wu , Y. Li , M. Kong , Z. Kang , B. Zuoyuan , and Z. Yang . 2022. “TFRC Upregulation Promotes Ferroptosis in CVB3 Infection via Nucleus Recruitment of Sp1.” Cell Death & Disease 13(7): 592. 10.1038/s41419-022-05027-w. 35821227 PMC9276735

[34] Meng, Z. , H. Liang , J. Zhao , J. Gao , C. Liu , X. Ma , J. Liu , et al. 2021. “HMOX1 Upregulation Promotes Ferroptosis in Diabetic Atherosclerosis.” Life Sciences 284: 119935. 10.1016/j.lfs.2021.119935. 34508760

[35] Dong, H. , Y. Xia , S. Jin , C. Xue , Y. Wang , R. Hu , and H. Jiang . 2021. “Nrf2 Attenuates Ferroptosis‐Mediated IIR‐ALI by Modulating TERT and SLC7A11.” Cell Death & Disease 12(11): 1027. 10.1038/s41419-021-04307-1. 34716298 PMC8556385

[36] Miao, Y. , Y. Chen , F. Xue , K. Liu , B. Zhu , J. Gao , J. Yin , C. Zhang , and G. Li . 2022. “Contribution of Ferroptosis and GPX4's Dual Functions to Osteoarthritis Progression.” EBioMedicine 76: 103847. 10.1016/j.ebiom.2022.103847. 35101656 PMC8822178

[37] Guiney, S. J. , P. A. Adlard , A. I. Bush , D. I. Finkelstein , and S. Ayton . 2017. “Ferroptosis and Cell Death Mechanisms in Parkinson's Disease.” Neurochemistry International 104: 34–48. 10.1016/j.neuint.2017.01.004. 28082232

