# Supplementary material for: Tert‐butyl hydroperoxide induces trabecular meshwork cells injury through ferroptotic cell death
Source: J Cell Commun Signal. 2024 Aug 28;18(3):e12050. doi: 10.1002/ccs3.12050 (PMC11544637; doi:10.1002/ccs3.12050)
Supplement: Supplementary file 1 — Table S1 [file CCS3-18-e12050-s002.docx]

| **Gene** | **Forward sequences (5’-3’)** | **Reverse sequences (5’-3’)** |
| --- | --- | --- |
| *β-actin* | CATGTACGTTGCTATCCAGGC | CTCCTTAATGTCACGCACGAT |
| *SLC7A11* | TGTGTGGGGTCCTGTCACTA | CAGTAGCTGCAGGGCGTATT |
| *HMOX1* | AAGACTGCGTTCCTGCTCAAC | AAAGCCCTACAGCAACTGTCG |
| *GPX4* | GGCAAGGGCATCCTGGGAAATG | CTTCACCACGCAGCCGTTCTT |
| *TFRC* | GCAGGAACCGAGTCTCCAGTGA | CGGTGAAGTCTGTGCTGTCCAG |

**Table S1.** Detailed information of primers
