# Supplementary material for: Tert‐butyl hydroperoxide induces trabecular meshwork cells injury through ferroptotic cell death
Source: J Cell Commun Signal. 2024 Aug 28;18(3):e12050. doi: 10.1002/ccs3.12050 (PMC11544637; doi:10.1002/ccs3.12050)
Supplement: Supplementary file 2 — Table S2 [file CCS3-18-e12050-s001.docx]

| **Antibody Name** | **Source** | **Catalog number** | **Dilution** |
| --- | --- | --- | --- |
| β-actin | Santa Cruz Biotechnology | SC-47778 | 1:1000 |
| SLC7A11 | Abcam | Ab307601 | 1:1000 |
| HMOX1 | Proteintech | 10701-1-AP | 1:1000 |
| GPX4 | Abcam | Ab125066 | 1:4000 |
| TFRC | Thermo | 13-6800 | 1:1000 |

**Table S2.** Detailed information of antibodies
